# Formation of Arbitrary Patterns in Ultraviolet Cured Polymer Film via Electrohydrodynamic Patterning

**DOI:** 10.1155/2014/840497

**Published:** 2014-02-24

**Authors:** Xin Li, Yucheng Ding, Jinyou Shao, Hongmiao Tian, Hongzhong Liu

**Affiliations:** ^1^State Key Laboratory for Manufacturing Systems Engineering, Xi'an Jiaotong University, Xi'an, Shaanxi 710049, China; ^2^College of Telecommunications and Information Engineering, Nanjing University of Posts and Telecommunications, Nanjing, Jiangsu 210023, China

## Abstract

Electrohydrodynamic patterning of arbitrary patterns is achieved by optimizing the critical parameters (applied voltage and spacer height). The applied voltage has a great influence on the fidelity of L-shaped line structures with different sizes. The L-shaped line structures with high fidelity are obtained by using the moderate applied voltage. The spacer height has a great influence on the fidelity of square structures with different sizes. The square structures with high fidelity are obtained by using the low height spacer. The multi-field coupling transient finite element simulation demonstrates that the lack of polymer owing to the high height spacer leads to the formation of defects.

## 1. Introduction

Polymer patterning has a wide range of applications in optoelectronics, biotechnology, novel sensors, actuators, and so on [1–3]. Electrohydrodynamic (EHD) patterning technique to fabricate micro-/nanostructures in thin polymer film has been developed in recent years [4–10]. EHD patterning is achieved by utilizing the interaction of an electric field with an interface between polymer film and another media of different permittivity [11]. The flat polymer film has the surface waves with small amplitude in the initial phase of EHD patterning. Then the amplitude of surface waves increases and the polymer film evolves into micro-/nanostructures.

The shape of structures formed in EHD patterning depends on the topography of template. The flat template produces the self-assembled pillars with hexagonal arrangement [6, 12, 13]. The periodicity of pillars is equal to the most unstable wavelength *λ* derived by the linear stability theory. *λ* is qualitatively determined by the surface tension of polymer, the polymer viscosity, and the electrostatic pressure [4, 11]. The influence of these parameters on the size and homogeneity of self-assembled pillars has been extensively analyzed in recent years [12, 14, 15]. Schäffer et al. and Chou and Zhuang and Deshpande et al. have shown the pillars with different size on large area by changing these parameters, respectively [5, 6, 16]. Zhuang used the flat template to induce the periodic pillars to fabricate an organic light emitting diode (OLED) with better performance [17].

The structures produced by the topographic template attract more attention from researcher [5, 18–20]. The electric field is laterally modulated by the template patterns. Researchers reported a lot of studies involving the structures with periodic patterns fabricated by EHD patterning [18, 19, 21]. Schäffer et al. and Chou et al. fabricated the periodic line gratings with submicron scale by using thermoplastic polymer, respectively [5, 20]. Goldberg-Oppenheimer and Steiner used a thermoplastic polymer with an extremely low viscosity to reduce the experimental time [22]. The researchers then developed the application devices by using the structures with periodic patterns fabricated by EHD patterning. Lee et al. fabricated the microlens array (periodic hemispherical) [23]. Hin et al. fabricated the optical waveguide by using template with line grating structures [24].

There is still lack of the research about EHD technique using template with arbitrary structures. Only a few researchers have paid attention to this field. Chou et al. created the structures with the pattern of the word “PRINCETON” with 3 *μ*m line width [20]. Harkema obtained the structures with the star patterns and observed the forming process [18]. He reported that the self-assembled pillars formed initially and then the pillars coalesced into the structures with the star patterns laterally. The forming mechanism of arbitrary structures remains elusive, and the demanding experimental requirements also hinder the development of fabrication of the structures with arbitrary patterns. The influence of processing parameters on the replication fidelity still needs further study. Furthermore, the line width and patterns of structures on one sample were uniform in previous work. The formation process of structures with significantly varying line width and complex patterns in EHD patterning requires a deeper understanding by means of experimental and theoretical studies.

In this paper, we presented the fabrication of the structures with different patterns and sizes on one sample and studied the influence of the critical parameters (the applied voltage and the spacer height) on the replication fidelity. The template patterns used in this paper can be divided into two categories: the patterns with aspect ratio of much larger than one such as the line patterns and the patterns with aspect ratio close to one such as square patterns. The two categories of patterns can basically cover most of the patterns in micro-/nanomanufacturing.

We show that the applied voltage has a great influence on the fidelity of structures of L-shaped line patterns with different size, and the spacer height has a great influence on the fidelity of structures of square patterns with different size. The multifield coupling simulation for the EHD patterning process is carried out.

## 2. Experimental

**Figure 1:**
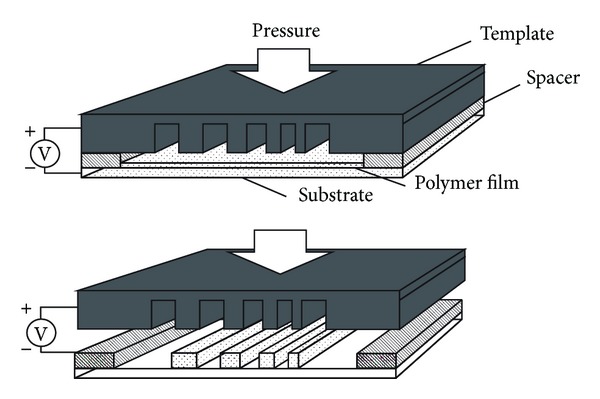
The schematic diagram of EHD patterning using the template with arbitrary patterns.

Figure 1 shows the schematic diagram of EHD patterning using the template with arbitrary patterns. The parallel plate capacitor geometry in the experiment setup was composed of two plates, a patterned template and a conductive transparent substrate. The template with arbitrary pattern based on silicon was fabricated by photolithography and etching. The patterns created the spatially modulated electric field. The surfactant with a low surface energy was coated on the template to facilitate the template-sample separation after the structures formation. Because the polymer used in our experiments was ultraviolet cured, the substrate needed to be transparent to let UV light through. The transparent substrate was glass slide covered with a conductive layer of indium tin oxide (ITO) with nanoscale thickness. The polymer was the ultraviolet cured polymer, Ormostamp (Micro Resist Technology, Germany). The dynamic viscosity of Ormostamp was between 0.41–0.46 Pa*∙*s. Its relative permittivity was 4.5 measured by a dielectric constant meter (BI-870, Brookhaven Instruments Corp., USA). The solvent Ormothin (Micro Resist Technology, Germany) was added to dilute Ormostamp. We mixed Ormostamp and Ormothin at mass ratio of 0.1 using a magnetic stirrer for 24 hours. The dynamic viscosity of mix with a mass ratio of 0.1 was 0.007 Pa*∙*s measured by a viscometer (DV-I Prime, Brookfield Engineering Laboratories, USA) with an adapter for low viscosity materials (Enhanced UL Adapter, Brookfield Engineering Laboratories, USA). The mix was spin-coated on a transparent substrate at 3000 rpm for 30 s. The film thickness was 400 nm measured by an ellipsometer (alpha-SE J. A. Woollam Co., Inc., USA). The air gap between the polymer film and the template patterns was controlled by an insulated spacer made of silicon dioxide. A press with 20 MPa was used to hold the gap distance unchanged. The voltage applied between template and substrate ranged from 20 V to 100 V. The spacer height of template ranged from 3 *μ*m to 4 *μ*m. The forming process of EHD patterning maintained 20 minutes. The structures were cured via UV light through the transparent substrate for 60 seconds.

## 3. Results and Discussions

There are two types of template patterns used in this paper. (a) The array of L-shaped line patterns: a single pattern has four L-shaped line lines. Their line widths are 2 *μ*m, 3 *μ*m, 4 *μ*m, and 5 *μ*m. (b) The array of square patterns: a single group of square patterns has eight square patterns. Their side lengths are 5 *μ*m, 10 *μ*m, 15 *μ*m, 20 *μ*m, 25 *μ*m, 30 *μ*m, 35 *μ*m, and 40 *μ*m.

**Figure 2:**
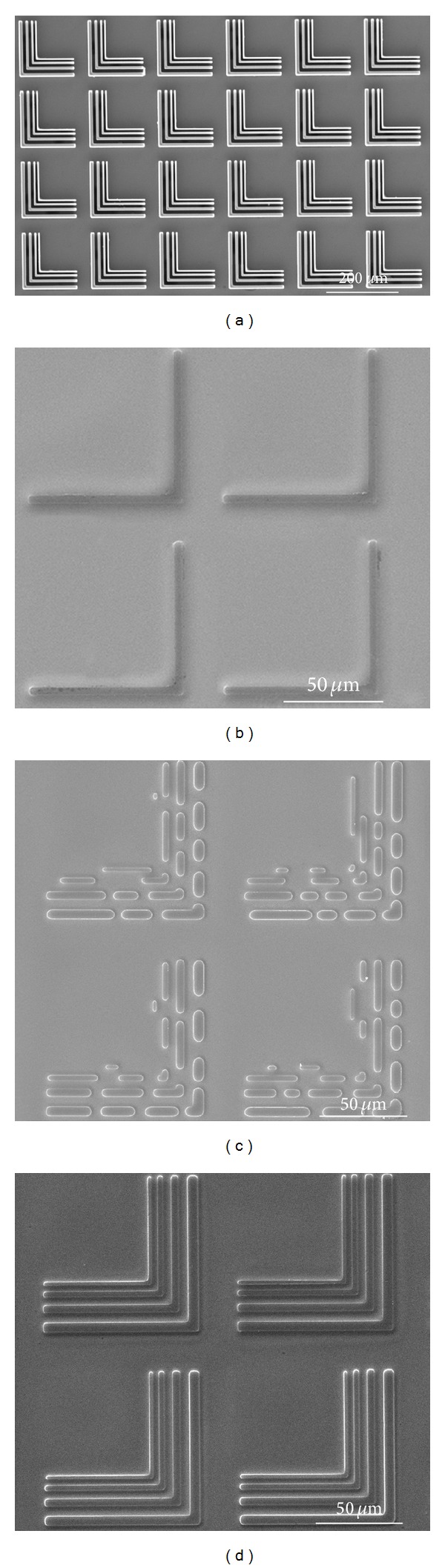
SEM images of the template with L-shaped line patterns and structures formed in EHD patterning. (a) The template, (b) the structures obtained with 30 V voltage, (c) the structures obtained with 80 V voltage, and (d) the structures obtained with 50 V voltage.

**Figure 3:**
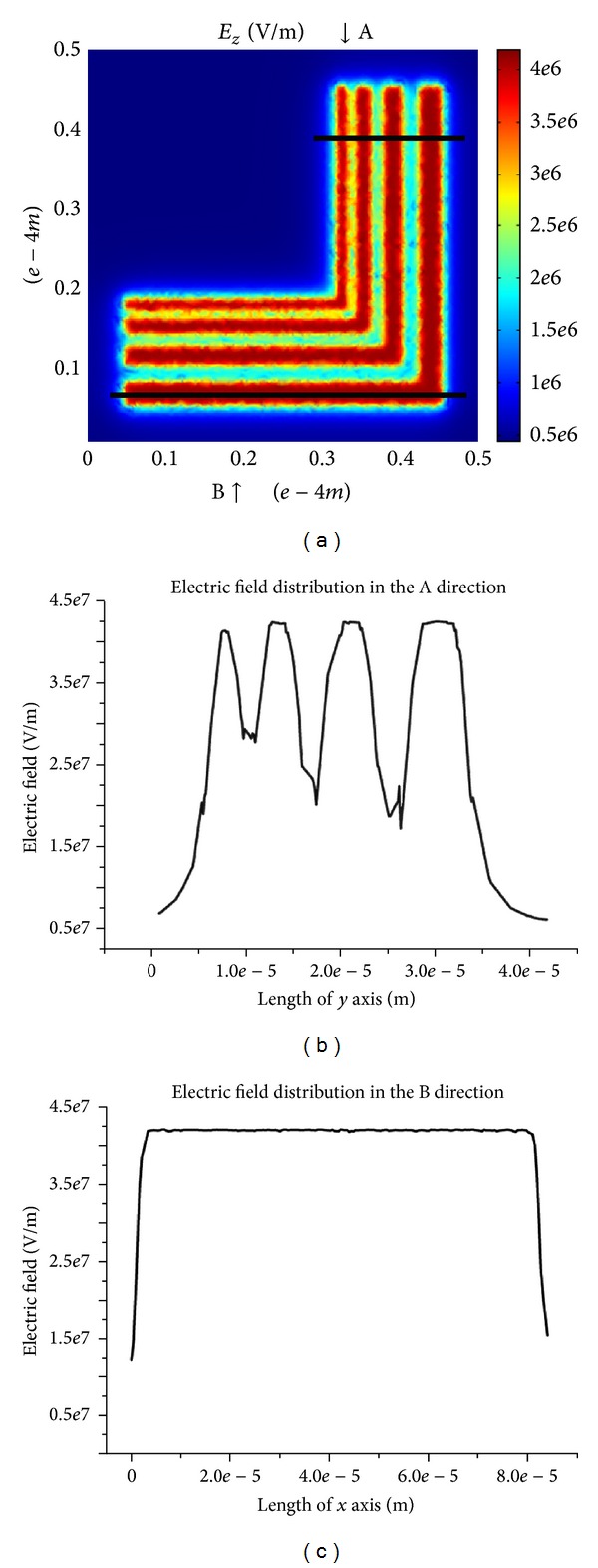
(a) *Z* component of the electric field on the surface of polymer film in initial phase, (b) A direction: the cross section of the L-shaped line, and (c) B direction: in axial direction of L-shaped line.

Figure 2 shows the template with L-shaped line patterns and the structures formed by different applied voltage. The applied voltage in Figure 3(b) is 30 V. The faithful structures of L-shaped line with 5 *μ*m line width formed in Figure 2(b) and the structures of the patterns with narrower line widths does not emerge. The applied voltage in Figure 2(c) is 80 V. The separated structures forms in Figure 2(c) and the line widths of these structures are consistent with those of the template patterns. The applied voltage in Figure 2(d) is 50 V. Figure 2(d) shows the faithful L-shaped structures with different line widths. The applied voltage significantly influences the replication fidelity. The moderate applied voltage is desired to obtain the faithful structures.

Theoretical of the EHD patterning is established to obtain the better understanding of the effects of the applied voltage on the replication fidelity. The driving force in EHD patterning deduces from the Maxwell stress tensor which represents the electric stress in the polymer film imposed by the electric field. The Maxwell tensor is expressed as follows [25]:

(1) $$\begin{array}{c} \overset{⃡}{T}=\varepsilon_{0}\varepsilon_{r}\vec{E}\vec{E}-\frac{1}{2}\varepsilon_{0}\varepsilon_{r}E^{2}\overset{⃡}{I}, \end{array}$$

where $\overset{\to }{E}$ is a vector variable denoting the electric field on the surface of the polymer film, *E* is a scalar variable denoting the electric intensity, *ε*_0_ is the permittivity of vacuum, *ε*_*r*_ is the relative permittivity of the polymer, and $\overset{⃡}{I}$ is the one tensor.

We assume that the polymer film located in *x*-*y* plane and the evolution process of EHD patterning are influenced by *z* component of the electric field which is in normal direction of polymer film. The driving force in EHD patterning can be transformed to the electrostatic pressure determined by *z* component of the electric field:

(2) $$\begin{array}{c} p_{e}=-\frac{1}{2}\varepsilon_{0}\varepsilon_{p}\left(\varepsilon_{p}-1\right)E_{z}^{2}, \end{array}$$

where *E*_*z*_ is a scalar variable denoting *z* component of the electric field on the surface of the polymer film and *ε*_*p*_ is the permittivity of polymer film.

The flow behavior of the polymer film as a continuum in an electric field can be expressed by the Navier-Stokes equation. The polymer used in our experiment is considered an incompressible Newtonian fluid. Therefore, the the Navier-Stokes equation is simplified as below:

(3) $$\begin{array}{c} \rho \frac{\partial \overset{\to }{v}}{\partial t}+\rho \overset{\to }{v}\cdot \nabla \overset{\to }{v}=-\nabla \left(p_{0}+p_{e}+p_{s}\right)+\mu \nabla^{2}\overset{\to }{v}+f, \end{array}$$

where *ρ* is the mass density of polymer, **v** is the velocity of polymer, *p*_0_ is atmosphere pressure, *p*_*s*_ is the pressure generated by surface tension, *μ* is the viscosity coefficient of polymer, and *f* is the volume driving force. $\mu \nabla^{2}\overset{\to }{v}$ and *f* could be neglected because their value is too small. *p*_0_ and *p*_*s*_ remain unchanged in the polymer film. Therefore, *p*_*e*_ determines the evolution process of EHD patterning. Additionally, the flow velocity of the polymer in *x*-*y* plane can be obtained by simplifying (3) according to the previous research:

(4) $$\begin{array}{c} u=\frac{1}{2\mu }z\left(z-2h\right)\Delta p, \end{array}$$

where *u* is a scalar variable denoting the flow velocity of polymer in *x*-*y* plane, *h* is the thickness of the polymer film in initial phase, *z* is the thickness of the polymer film in real time, and Δ*p* is the electrostatic pressure difference between the polymer under the template protrusion patterns and the polymer under the template cavity area. Equation (4) shows that the flow behavior in EHD patterning was strongly influenced by Δ*p*.

We utilize the commercial FEM software (COMSOL Multiphysics) to analyze the electric field distribution on the surface of polymer film in the initial phase of EHD patterning. The applied voltage in simulation is set to 50 V.  Figure 3(a) shows the *z* component of the electric field distribution on the surface of polymer film in initial phase. The electric field under the L-shaped line pattern is stronger than that under the template cavity. The electric field is divided into two parts: A direction: the cross section of the L-shaped line, B direction: in axial direction of L-shaped line.

The electric field in A direction is strongly modulated by the lines. We set Δ*p* to the difference between the electrostatic pressure under the line patterns with different line widths and the electrostatic pressure under the template cavity in right side of these patterns:

(5) $$\begin{array}{c} \Delta p=-\frac{1}{2}\varepsilon_{0}\varepsilon_{p}\left(\varepsilon_{p}-1\right)\left(E_{p}^{2}-E_{l}^{2}\right), \end{array}$$

where *E*_*p*_ is a scalar variable denoting *z* component of the electric field on the surface of the polymer film under the line patterns and *E*_*l*_ is a scalar variable denoting *z* component of the electric field on the surface of the polymer film under the template cavity in right side of these patterns.

**Figure 4:**
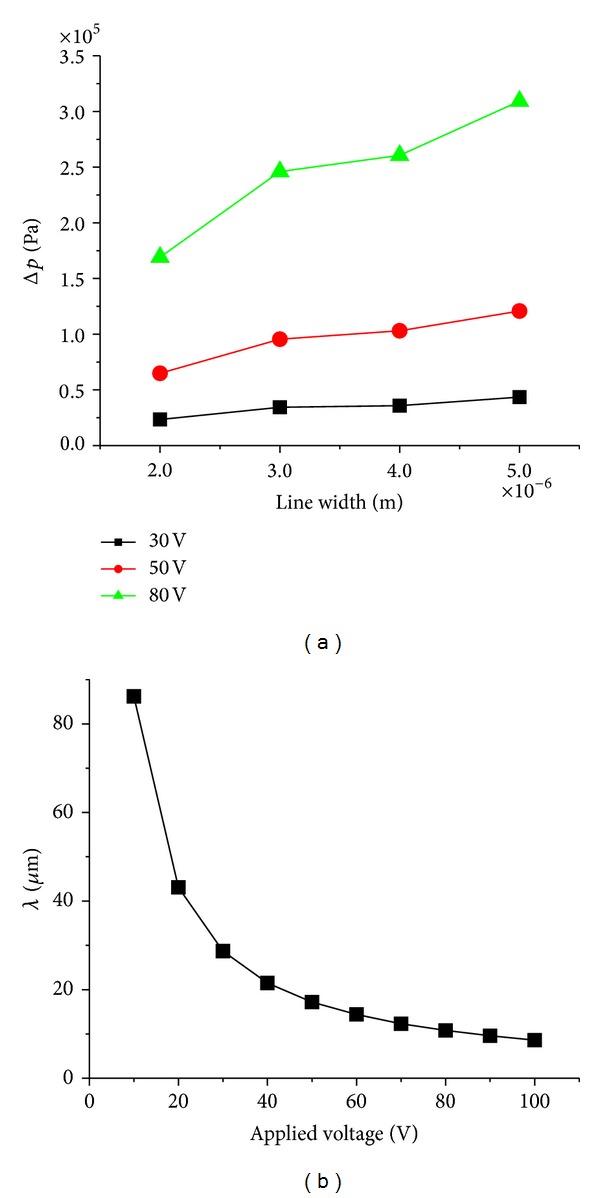
(a) Δ*p* with different applied voltage, (b) *λ* the period of the separated structures with different applied voltage.

The template patterns with broader line width generate the accumulation of the polymer film in A direction more easily. Δ*p* of the template patterns with different line width increases under higher voltage. The experimental result in Figure 2 supports the analysis that Δ*p* mainly determines the replication fidelity in cross section of the template patterns. The homogeneous electric field in B direction induced the separated periodic structures according to the linear stability analysis theory. The stronger electric field is more conductive to the formation of the separated structures.  Figure 4(b) shows *λ* the period of the separated structures with different applied voltage. The period of the separated structures gets larger with the reduction of applied voltage. The separated structures with large size tend to coalesce into an integral structure. The formation of separated structures with small size is generated by larger applied voltage. Figure 2(c) shows the separated structures.

**Figure 5:**
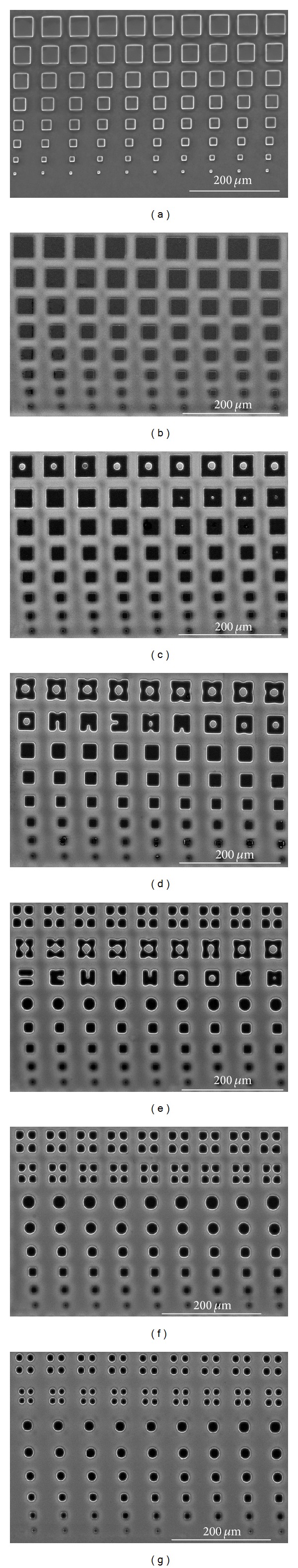
SEM images of the template with square patterns and structures formed in EHD patterning. (a) The template with square patterns, (b) the structures obtained in experiment with 3.0 *μ*m spacer height, (c) with 3.2 *μ*m spacer height, (d) with 3.4 *μ*m spacer height, (e) with 3.6 *μ*m spacer height, (f) with 3.8 *μ*m spacer height, and (g) with 4.0 *μ*m spacer height.

Figure 5 shows the template with square patterns and the structures formed with different spacer height. The structures in Figure 5(b) have high fidelity. The central part of structures with 40 *μ*m and 35 *μ*m side lengths in Figure 5(c) has cavity defects. The cavity defects of structures with the 40 *μ*m side lengths become larger in Figure 5(d). The spacer height has a significant impact on the fidelity of structures obtained in EHD patterning. The smaller spacer height contributes to the formation of the structures with high fidelity. The structures with smaller size get the higher fidelity when the spacer height is constant.

We carry out the multifield coupling simulation for the EHD patterning process of the array of square patterns with different size. The geometric model of the simulation is set to be a two-dimensional cross-section model through the center of the square patterns. The simulation uses a uniform phase field model including three control equations: Gauss equation for electrical field, Navier-Stokes equation for flow field, and Cahn-Hilliard equation for phase field. The phase field model uses a boundary layer to replace the liquid-gas interface. There is no strict distinction between the polymer and air which are treated as a total fluid in the simulation process. The change of the gas-liquid interface is obtained by tracking the phase function *ϕ*. *ϕ* is a basic phase function of position vector describing the types of substance. The range of *ϕ* is from 0 to 1 in phase field. 0 represents the polymer, and 1 represents the air in our model. 0.5 represents the interface between the two substances. We analyze the experiment with the lowest spacer (3.0 *μ*m) and the experiment with the highest spacer (4.0 *μ*m).

**Figure 6:**
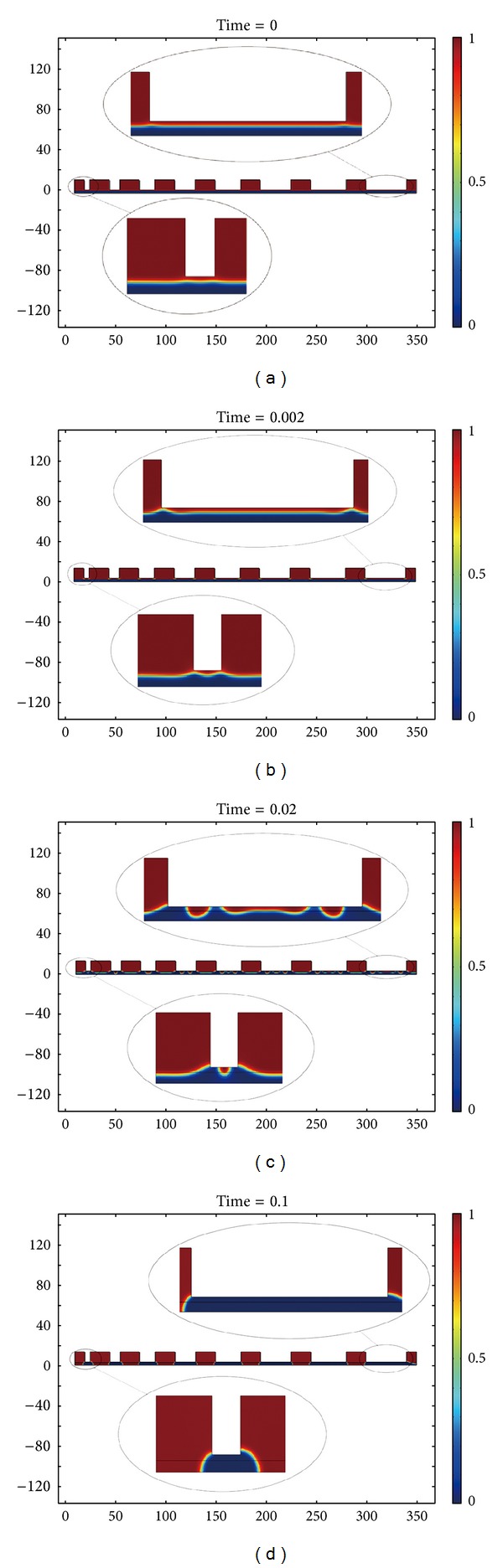
The evolution process of EHD patterning experiment with the lowest spacer (3.0 *μ*m). (a) The initial stage of EHD patterning, (b) and (c) the evolutionary stage of EHD patterning, and (d) the final stage of EHD patterning.

Figure 6 shows the evolution process of EHD patterning with the 3.0 *μ*m spacer. The blue region is the polymer; the red region is the air. The white protrusion is the cross section of template. We enlarge the shape of interface at the template patterns with 40 *μ*m and the template patterns with 5 *μ*m to contrast the size effect on EHD patterning. The polymer always flows upward to the template patterns and forms the structures with high fidelity.  Figure 6(a) shows the initial stage of EHD patterning. The electrical field is larger at the edge of template patterns owing to larger electrostatic pressure. The polymer closing to the edge of template patterns flow upward firstly, and then the flow behavior extends to the polymer closing to the center of template patterns in Figures 6(b) and 6(c).  Figure 6(d) shows the final stage of EHD patterning that the polymer under the template patterns merges to form the structures with high fidelity.

**Figure 7:**
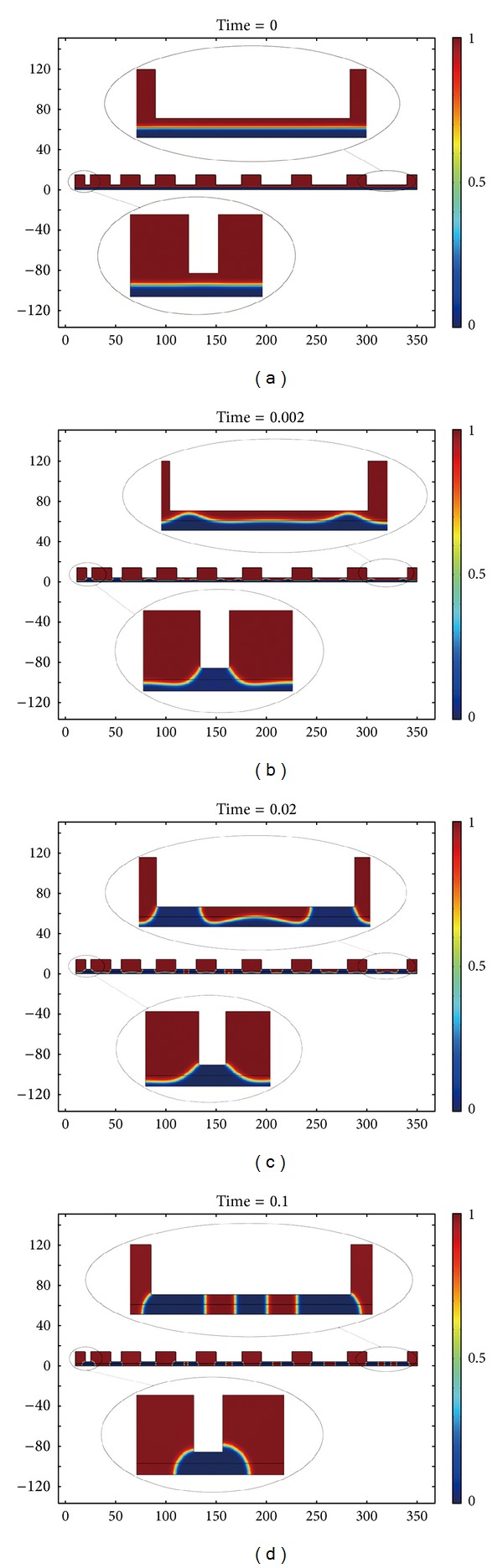
The evolution process of EHD patterning experiment with the highest spacer (4.0 *μ*m). (a) The initial stage of EHD patterning, (b) and (c) the evolutionary stage of EHD patterning, and (d) the final stage of EHD patterning.

Figure 7 shows the evolution process of EHD patterning experiment with 4.0 *μ*m spacer. In condition that the spacer height is increased, the polymer under the template patterns with small size could still merge to form the structures with high fidelity. However, the higher spacer generates a greater impact on the polymer under the template patterns with large size. The replication for the template patterns with large size needs more polymer than that for the template patterns with small size. The lack of polymer results in the fact that the separated pillars could not merge to the structures with high fidelity.

## 4. Conclusions

We demonstrate that the optimization of applied voltage can improve the replication fidelity of the structures with line patterns having different size. The constant voltage is applied on the patterned template to generate the spatially modulated electric field, and the flow behavior of the polymer film in experiment is significantly influenced by Δ*p*. We could obtain the structures of line patterns with high fidelity by using the moderate applied voltage. The optimization of spacer height can improve the replication fidelity of the structures with square patterns having different size. The high fidelity structures could be obtained under the low height spacer. The work in this paper has important implications for the capability and versatility of EHD patterning of ultraviolet cured polymer film.
